# Methyl 2,2-diphenyl-2-(prop-2-yn-1-yl­oxy)acetate

**DOI:** 10.1107/S1600536812007982

**Published:** 2012-02-29

**Authors:** H. P. Sumathi, Ulrich Flörke, H. S. Yathirajan, A. S. Dayananda, A. R. Ramesha

**Affiliations:** a Department of Studies in Chemistry, University of Mysore, Manasagangotri, Mysore 570 006, India; b Department Chemie, Fakultät für Naturwissenschaften, Universität Paderborn, Warburgerstrasse 100, D-33098 Paderborn, Germany; c R. L. Fine Chemicals, Bangalore 560 064, India

## Abstract

The mol­ecular structure of the title compound, C_18_H_16_O_3_, exhibits a new *R*_2_–C(COOMe)(OCH_2_CCH) group. The C—O—C—C torsion angle is 153.3 (1)°. The dihedral angles are 79.89 (5)° between phen­yl/phenyl planes, and 73.13 (5) and 79.05 (8)° for the two COOMe/phenyl plane pairs.

## Related literature

For related literature on the background of this work, see: Ferguson *et al.* (1995 ▶); Ohkuma *et al.* (2000 ▶). For related structures, see: Narayanan *et al.* (2011 ▶); Shah *et al.* (2011 ▶); Siddaraju *et al.* (2010 ▶); Zhang *et al.* (2008 ▶); Zhang *et al.* (2011 ▶).
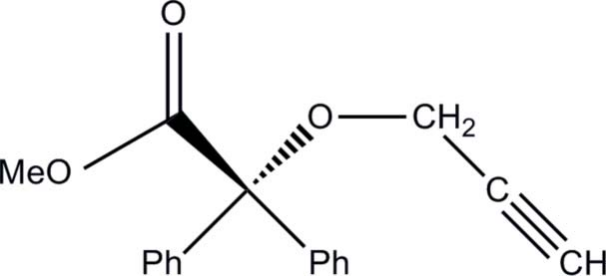


## Experimental

### 

#### Crystal data

C_18_H_16_O_3_
*M* *_r_* = 280.31
Monoclinic, 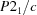

*a* = 12.6771 (17) Å
*b* = 9.2410 (13) Å
*c* = 12.7055 (18) Å
β = 93.090 (3)°
*V* = 1486.3 (4) Å^3^
*Z* = 4
Mo *K*α radiation
μ = 0.09 mm^−1^
*T* = 130 K
0.37 × 0.22 × 0.10 mm

#### Data collection

Bruker SMART APEX diffractometer
Absorption correction: multi-scan (*SADABS*; Sheldrick, 2004 ▶) *T*_min_ = 0.969, *T*_max_ = 0.992
13808 measured reflections
3545 independent reflections
2623 reflections with *I* > 2σ(*I*)
*R*_int_ = 0.045

#### Refinement

*R*[*F* ^2^ > 2σ(*F* ^2^)] = 0.048
*wR*(*F* ^2^) = 0.117
*S* = 1.02
3545 reflections
195 parameters
H atoms treated by a mixture of independent and constrained refinement
Δρ_max_ = 0.33 e Å^−3^
Δρ_min_ = −0.19 e Å^−3^

### 

Data collection: *SMART* (Bruker, 2002 ▶); cell refinement: *SAINT* (Bruker, 2002 ▶); data reduction: *SAINT*; program(s) used to solve structure: *SHELXTL* (Sheldrick, 2008 ▶); program(s) used to refine structure: *SHELXTL*; molecular graphics: *SHELXTL*; software used to prepare material for publication: *SHELXTL* and local programs.

## Supplementary Material

Crystal structure: contains datablock(s) I, global. DOI: 10.1107/S1600536812007982/nc2267sup1.cif

Structure factors: contains datablock(s) I. DOI: 10.1107/S1600536812007982/nc2267Isup2.hkl

Supplementary material file. DOI: 10.1107/S1600536812007982/nc2267Isup3.cml

Additional supplementary materials:  crystallographic information; 3D view; checkCIF report

## Figures and Tables

**Fig. 1.**
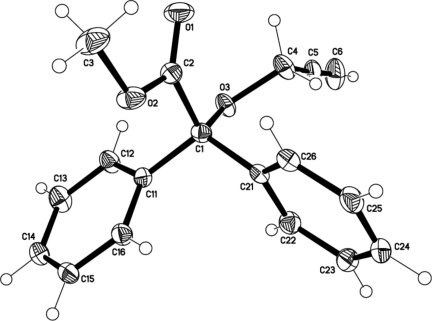
Molecular structure with labeling and displacement ellipsoids drawn at the 50% probability level.

## supplementary crystallographic information

### Experimental

The title compound was obtained as a gift sample from R. L. Fine Chem., Bengaluru, India. X-ray quality crystals were obtained from toluene by slow evaporation (m.p. 318 K).

### Refinement

H atoms were clearly identified in difference syntheses, idealized and refined riding on the C atoms with C—H = 0.95–0.99 Å, and with isotropic displacement parameters *U*_iso_(H) = 1.2U(C_eq_) or 1.5U(–CH_3_ H atoms). All CH_3_ H atoms were allowed to rotate but not to tip. H6 was refined freely.

### Crystal data

**Table:**

C_18_H_16_O_3_	*F*(000) = 592
*M**_r_* = 280.31	*D*_x_ = 1.253 Mg m^−^^3^
Monoclinic, *P*2_1_/*c*	Mo *K*α radiation, λ = 0.71073 Å
Hall symbol: -P 2ybc	Cell parameters from 1710 reflections
*a* = 12.6771 (17) Å	θ = 2.7–23.4°
*b* = 9.2410 (13) Å	µ = 0.09 mm^−^^1^
*c* = 12.7055 (18) Å	*T* = 130 K
β = 93.090 (3)°	Prism, colourless
*V* = 1486.3 (4) Å^3^	0.37 × 0.22 × 0.10 mm
*Z* = 4	

### Data collection

**Table:**

Bruker SMART APEX diffractometer	3545 independent reflections
Radiation source: sealed tube	2623 reflections with *I* > 2σ(*I*)
Graphite monochromator	*R*_int_ = 0.045
φ and ω scans	θ_max_ = 27.9°, θ_min_ = 1.6°
Absorption correction: multi-scan (*SADABS*; Sheldrick, 2004)	*h* = −14→16
*T*_min_ = 0.969, *T*_max_ = 0.992	*k* = −12→12
13808 measured reflections	*l* = −16→16

### Refinement

**Table:**

Refinement on *F*^2^	Primary atom site location: structure-invariant direct methods
Least-squares matrix: full	Secondary atom site location: difference Fourier map
*R*[*F*^2^ > 2σ(*F*^2^)] = 0.048	Hydrogen site location: difference Fourier map
*wR*(*F*^2^) = 0.117	H atoms treated by a mixture of independent and constrained refinement
*S* = 1.02	*w* = 1/[σ^2^(*F*_o_^2^) + (0.0487*P*)^2^ + 0.3727*P*] where *P* = (*F*_o_^2^ + 2*F*_c_^2^)/3
3545 reflections	(Δ/σ)_max_ < 0.001
195 parameters	Δρ_max_ = 0.33 e Å^−^^3^
0 restraints	Δρ_min_ = −0.19 e Å^−^^3^

### Special details

**Table:**

Geometry. All e.s.d.'s (except the e.s.d. in the dihedral angle between two l.s. planes) are estimated using the full covariance matrix. The cell e.s.d.'s are taken into account individually in the estimation of e.s.d.'s in distances, angles and torsion angles; correlations between e.s.d.'s in cell parameters are only used when they are defined by crystal symmetry. An approximate (isotropic) treatment of cell e.s.d.'s is used for estimating e.s.d.'s involving l.s. planes.
Refinement. Refinement of *F*^2^ against ALL reflections. The weighted *R*-factor *wR* and goodness of fit *S* are based on *F*^2^, conventional *R*-factors *R* are based on *F*, with *F* set to zero for negative *F*^2^. The threshold expression of *F*^2^ > σ(*F*^2^) is used only for calculating *R*-factors(gt) *etc*. and is not relevant to the choice of reflections for refinement. *R*-factors based on *F*^2^ are statistically about twice as large as those based on *F*, and *R*-factors based on ALL data will be even larger.

### Fractional atomic coordinates and isotropic or equivalent isotropic displacement parameters (Å^2^)

**Table:**

	*x*	*y*	*z*	*U*_iso_*/*U*_eq_	
O1	0.79582 (9)	0.45794 (12)	0.41949 (10)	0.0378 (3)	
O2	0.66203 (9)	0.37609 (11)	0.51072 (9)	0.0296 (3)	
O3	0.78724 (8)	0.22289 (11)	0.28970 (8)	0.0247 (3)	
C1	0.72467 (12)	0.21809 (15)	0.38023 (11)	0.0203 (3)	
C2	0.73393 (12)	0.36505 (15)	0.43855 (12)	0.0233 (3)	
C3	0.66088 (16)	0.51182 (17)	0.56825 (15)	0.0393 (5)	
H3A	0.7287	0.5250	0.6078	0.059*	
H3B	0.6038	0.5100	0.6173	0.059*	
H3C	0.6494	0.5920	0.5185	0.059*	
C4	0.90005 (12)	0.22196 (19)	0.31004 (13)	0.0299 (4)	
H4A	0.9188	0.1639	0.3738	0.036*	
H4B	0.9261	0.3219	0.3222	0.036*	
C5	0.94840 (13)	0.15941 (18)	0.21894 (14)	0.0302 (4)	
C6	0.99076 (16)	0.1114 (2)	0.14631 (16)	0.0432 (5)	
H6	1.0260 (19)	0.068 (3)	0.0870 (19)	0.073 (7)*	
C11	0.60998 (11)	0.20274 (15)	0.33670 (11)	0.0199 (3)	
C12	0.57858 (12)	0.25763 (17)	0.23856 (13)	0.0271 (4)	
H12A	0.6288	0.3037	0.1969	0.032*	
C13	0.47418 (13)	0.24566 (19)	0.20079 (13)	0.0326 (4)	
H13A	0.4534	0.2830	0.1332	0.039*	
C14	0.40033 (13)	0.17983 (17)	0.26091 (13)	0.0291 (4)	
H14A	0.3292	0.1700	0.2343	0.035*	
C15	0.43066 (12)	0.12818 (17)	0.36029 (13)	0.0279 (4)	
H15A	0.3798	0.0853	0.4028	0.033*	
C16	0.53494 (12)	0.13889 (16)	0.39776 (13)	0.0247 (3)	
H16A	0.5554	0.1023	0.4657	0.030*	
C21	0.76008 (11)	0.09067 (15)	0.45070 (11)	0.0198 (3)	
C22	0.76063 (12)	−0.04599 (16)	0.40361 (13)	0.0255 (3)	
H22A	0.7343	−0.0573	0.3327	0.031*	
C23	0.79918 (13)	−0.16498 (17)	0.45948 (14)	0.0307 (4)	
H23A	0.7992	−0.2575	0.4269	0.037*	
C24	0.83784 (13)	−0.14904 (17)	0.56317 (14)	0.0312 (4)	
H24A	0.8648	−0.2305	0.6015	0.037*	
C25	0.83705 (13)	−0.01480 (18)	0.61038 (13)	0.0294 (4)	
H25A	0.8628	−0.0040	0.6815	0.035*	
C26	0.79855 (12)	0.10528 (16)	0.55403 (12)	0.0239 (3)	
H26A	0.7988	0.1977	0.5868	0.029*	

### Atomic displacement parameters (Å^2^)

**Table:**

	*U*^11^	*U*^22^	*U*^33^	*U*^12^	*U*^13^	*U*^23^
O1	0.0357 (7)	0.0252 (6)	0.0539 (8)	−0.0100 (5)	0.0158 (6)	−0.0027 (5)
O2	0.0363 (7)	0.0233 (6)	0.0304 (6)	−0.0054 (5)	0.0122 (5)	−0.0064 (4)
O3	0.0162 (5)	0.0372 (6)	0.0210 (6)	0.0001 (4)	0.0048 (4)	0.0035 (4)
C1	0.0200 (8)	0.0221 (7)	0.0192 (7)	−0.0014 (6)	0.0044 (6)	0.0010 (6)
C2	0.0222 (8)	0.0225 (7)	0.0252 (8)	−0.0003 (6)	0.0006 (6)	0.0029 (6)
C3	0.0522 (12)	0.0246 (8)	0.0424 (11)	−0.0033 (8)	0.0146 (9)	−0.0111 (7)
C4	0.0174 (8)	0.0434 (10)	0.0292 (9)	−0.0015 (7)	0.0042 (7)	0.0028 (7)
C5	0.0207 (8)	0.0364 (9)	0.0338 (9)	0.0061 (7)	0.0034 (7)	0.0106 (7)
C6	0.0339 (11)	0.0559 (12)	0.0407 (11)	0.0179 (9)	0.0090 (9)	0.0058 (9)
C11	0.0183 (8)	0.0195 (7)	0.0222 (8)	0.0014 (5)	0.0029 (6)	−0.0005 (5)
C12	0.0207 (8)	0.0349 (8)	0.0259 (8)	−0.0001 (6)	0.0036 (7)	0.0052 (7)
C13	0.0283 (9)	0.0455 (10)	0.0237 (8)	0.0029 (7)	−0.0016 (7)	0.0074 (7)
C14	0.0191 (8)	0.0317 (8)	0.0361 (9)	0.0006 (6)	−0.0028 (7)	0.0011 (7)
C15	0.0195 (8)	0.0289 (8)	0.0355 (9)	−0.0009 (6)	0.0042 (7)	0.0077 (7)
C16	0.0211 (8)	0.0271 (8)	0.0261 (8)	0.0005 (6)	0.0026 (6)	0.0065 (6)
C21	0.0143 (7)	0.0222 (7)	0.0231 (8)	−0.0017 (5)	0.0022 (6)	0.0003 (6)
C22	0.0241 (8)	0.0262 (8)	0.0260 (8)	0.0000 (6)	−0.0011 (7)	−0.0031 (6)
C23	0.0254 (9)	0.0236 (8)	0.0430 (10)	0.0013 (6)	0.0001 (8)	−0.0024 (7)
C24	0.0214 (8)	0.0296 (8)	0.0418 (10)	0.0013 (6)	−0.0044 (7)	0.0100 (7)
C25	0.0231 (9)	0.0364 (9)	0.0277 (9)	−0.0020 (7)	−0.0068 (7)	0.0050 (7)
C26	0.0194 (8)	0.0263 (7)	0.0260 (8)	−0.0031 (6)	−0.0001 (6)	−0.0008 (6)

### Geometric parameters (Å, º)

**Table:**

O1—C2	1.1965 (18)	C12—H12A	0.9500
O2—C2	1.3312 (18)	C13—C14	1.381 (2)
O2—C3	1.4522 (18)	C13—H13A	0.9500
O3—C1	1.4327 (17)	C14—C15	1.385 (2)
O3—C4	1.4396 (18)	C14—H14A	0.9500
C1—C21	1.532 (2)	C15—C16	1.384 (2)
C1—C11	1.534 (2)	C15—H15A	0.9500
C1—C2	1.548 (2)	C16—H16A	0.9500
C3—H3A	0.9800	C21—C26	1.382 (2)
C3—H3B	0.9800	C21—C22	1.398 (2)
C3—H3C	0.9800	C22—C23	1.384 (2)
C4—C5	1.458 (2)	C22—H22A	0.9500
C4—H4A	0.9900	C23—C24	1.388 (2)
C4—H4B	0.9900	C23—H23A	0.9500
C5—C6	1.179 (3)	C24—C25	1.378 (2)
C6—H6	0.98 (2)	C24—H24A	0.9500
C11—C12	1.385 (2)	C25—C26	1.394 (2)
C11—C16	1.391 (2)	C25—H25A	0.9500
C12—C13	1.388 (2)	C26—H26A	0.9500
			
C2—O2—C3	116.02 (12)	C14—C13—C12	120.41 (15)
C1—O3—C4	116.32 (11)	C14—C13—H13A	119.8
O3—C1—C21	109.60 (11)	C12—C13—H13A	119.8
O3—C1—C11	105.57 (11)	C13—C14—C15	119.54 (15)
C21—C1—C11	112.45 (11)	C13—C14—H14A	120.2
O3—C1—C2	109.03 (11)	C15—C14—H14A	120.2
C21—C1—C2	112.47 (12)	C16—C15—C14	120.13 (15)
C11—C1—C2	107.43 (11)	C16—C15—H15A	119.9
O1—C2—O2	124.42 (14)	C14—C15—H15A	119.9
O1—C2—C1	124.47 (14)	C15—C16—C11	120.51 (14)
O2—C2—C1	111.08 (12)	C15—C16—H16A	119.7
O2—C3—H3A	109.5	C11—C16—H16A	119.7
O2—C3—H3B	109.5	C26—C21—C22	119.02 (13)
H3A—C3—H3B	109.5	C26—C21—C1	123.87 (13)
O2—C3—H3C	109.5	C22—C21—C1	116.90 (13)
H3A—C3—H3C	109.5	C23—C22—C21	120.55 (15)
H3B—C3—H3C	109.5	C23—C22—H22A	119.7
O3—C4—C5	108.41 (13)	C21—C22—H22A	119.7
O3—C4—H4A	110.0	C22—C23—C24	119.95 (15)
C5—C4—H4A	110.0	C22—C23—H23A	120.0
O3—C4—H4B	110.0	C24—C23—H23A	120.0
C5—C4—H4B	110.0	C25—C24—C23	119.87 (15)
H4A—C4—H4B	108.4	C25—C24—H24A	120.1
C6—C5—C4	177.6 (2)	C23—C24—H24A	120.1
C5—C6—H6	178.2 (14)	C24—C25—C26	120.22 (15)
C12—C11—C16	119.06 (14)	C24—C25—H25A	119.9
C12—C11—C1	120.77 (13)	C26—C25—H25A	119.9
C16—C11—C1	120.11 (13)	C21—C26—C25	120.38 (14)
C11—C12—C13	120.32 (15)	C21—C26—H26A	119.8
C11—C12—H12A	119.8	C25—C26—H26A	119.8
C13—C12—H12A	119.8		
			
C4—O3—C1—C21	−52.38 (16)	C11—C12—C13—C14	0.4 (3)
C4—O3—C1—C11	−173.72 (12)	C12—C13—C14—C15	1.3 (3)
C4—O3—C1—C2	71.12 (15)	C13—C14—C15—C16	−1.8 (2)
C3—O2—C2—O1	0.2 (2)	C14—C15—C16—C11	0.7 (2)
C3—O2—C2—C1	−178.05 (13)	C12—C11—C16—C15	1.1 (2)
O3—C1—C2—O1	−10.3 (2)	C1—C11—C16—C15	178.21 (13)
C21—C1—C2—O1	111.46 (17)	O3—C1—C21—C26	119.93 (15)
C11—C1—C2—O1	−124.27 (16)	C11—C1—C21—C26	−122.97 (15)
O3—C1—C2—O2	167.95 (11)	C2—C1—C21—C26	−1.5 (2)
C21—C1—C2—O2	−70.27 (16)	O3—C1—C21—C22	−54.88 (16)
C11—C1—C2—O2	54.00 (15)	C11—C1—C21—C22	62.22 (17)
C1—O3—C4—C5	153.28 (13)	C2—C1—C21—C22	−176.34 (13)
O3—C1—C11—C12	−28.55 (17)	C26—C21—C22—C23	−0.1 (2)
C21—C1—C11—C12	−148.02 (14)	C1—C21—C22—C23	175.02 (14)
C2—C1—C11—C12	87.70 (16)	C21—C22—C23—C24	−0.1 (2)
O3—C1—C11—C16	154.35 (13)	C22—C23—C24—C25	0.4 (2)
C21—C1—C11—C16	34.87 (18)	C23—C24—C25—C26	−0.6 (2)
C2—C1—C11—C16	−89.40 (15)	C22—C21—C26—C25	−0.2 (2)
C16—C11—C12—C13	−1.6 (2)	C1—C21—C26—C25	−174.87 (14)
C1—C11—C12—C13	−178.74 (14)	C24—C25—C26—C21	0.5 (2)

## References

[bb1] Bruker (2002). *SMART* and *SAINT* Bruker AXS Inc., Madison, Wisconsin, USA.

[bb2] Ferguson, G., Carroll, C. D., Glidewell, C., Zakaria, C. M. & Lough, A. J. (1995). *Acta Cryst.* B**51**, 367–377.

[bb3] Narayanan, P., Sethusankar, K., Ramachandiran, K. & Perumal, P. T. (2011). *Acta Cryst.* E**67**, o2658. 10.1107/S1600536811036907 PMC3201230 22058778

[bb4] Ohkuma, T., Koizumi, M., Ikehira, H., Yokozawa, T. & Noyori, R. (2000). *Org. Lett.* **2**, 659–662. 10.1021/ol9904139 10814403

[bb5] Shah, K., Raza Shah, M. & Ng, S. W. (2011). *Acta Cryst.* E**67**, o568. 10.1107/S1600536811003874 PMC3052146 21522331

[bb6] Sheldrick, G. M. (2004). *SADABS* University of Göttingen, Germany.

[bb7] Sheldrick, G. M. (2008). *Acta Cryst.* A**64**, 112–122. 10.1107/S0108767307043930 18156677

[bb8] Siddaraju, B. P., Yathirajan, H. S., Narayana, B., Ng, S. W. & Tiekink, E. R. T. (2010). *Acta Cryst.* E**66**, o2136. 10.1107/S1600536810029417 PMC3007446 21588424

[bb9] Zhang, W., Yao, L. & Tao, R.-J. (2008). *Acta Cryst.* E**64**, o307. 10.1107/S160053680706638X PMC2915354 21200870

[bb10] Zhang, C.-H., Zhao, J.-M. & Chen, B.-G. (2011). *Acta Cryst.* E**67**, o150.

